# Ambient Ozone and Emergency Department Visits for Cellulitis

**DOI:** 10.3390/ijerph7114078

**Published:** 2010-11-19

**Authors:** Mieczysław Szyszkowicz, Eugeniusz Porada, Gilaad G. Kaplan, Brian H. Rowe

**Affiliations:** 1 Population Studies Division, Health Canada, 269 Laurier Avenue, Ottawa, ON K1A 0K9, Canada; E-Mail: eporada@hotmail.com (E.P.); 2 Departments of Medicine and Community Health Sciences, University of Calgary, 2500 University Drive, Calgary, AB T2N 1N4, Canada; E-Mail: ggkaplan@ucalgary.ca (G.K.); 3 Department of Emergency Medicine, University of Alberta, 8440-112 Street, Edmonton, AB T6G 2B7, Canada; E-Mail: browe@ualberta.ca (B.R.); 4 School of Public Health, University of Alberta, 8440-112 Street, Edmonton, AB T6G 2B7, Canada

**Keywords:** cellulitis, skin infection, emergency department visit, ozone, bacteria, urban

## Abstract

Objectives were to assess and estimate an association between exposure to ground-level ozone and emergency department (ED) visits for cellulitis. All ED visits for cellulitis in Edmonton, Canada, in the period April 1992–March 2002 (N = 69,547) were examined. Case-crossover design was applied to estimate odds ratio (OR, and 95% confidence interval) per one interquartile range (IQR) increase in ozone concentration (IQR = 14.0 ppb). Delay of ED visit relating to exposure was probed using 0- to 5-day exposure lags. For all patients in the all months (January–December) and lags 0 to 2 days, OR = 1.05 (1.02, 1.07). For male patients during the cold months (October–March): OR = 1.05 (1.02, 1.09) for lags 0 and 2 and OR = 1.06 (1.02, 1.10) for lag 3. For female patients in the warm months (April–September): OR = 1.12 (1.06, 1.18) for lags 1 and 2. Cellulitis developing on uncovered (more exposed) skin was analyzed separately, observed effects being stronger. Cellulitis may be associated with exposure to ambient ground level ozone; the exposure may facilitate cellulitis infection and aggravate acute symptoms.

## 1. Introduction

Several studies have demonstrated that ozone (O_3_) adversely affects health [1–8] and is a risk factor for respiratory conditions such as asthma [9–11]. A growing body of research has linked ambient ozone to non respiratory conditions such as cardiac disease [11,12], appendicitis [13], headaches and migraines [14,15]. Cellulitis is an acute inflammation of deep skin caused most predominantly by infection from *staphylococcus aureus* and *streptococcus* species. The bacterium incubation period varies and can be several days or less than 24 hours. Deep and rapidly spreading infections develop into severe emergencies. In the healthy individual, cellulitis may be trigged by a break in the skin from trauma, animal bites, or other skin diseases. Moreover, certain groups are more susceptible such as those with venous stasis, diabetes mellitus and immuno-suppresion (e.g., human immunodeficiency virus or medical immuno-suppression from chemotherapy). Most often, however, a discernable risk factor is not identified [16,17]. We hypothesize that exposure to ozone may damage the skin through oxidative stress allowing bacteria to penetrate the skin.

In this study we explore the hypothesis that exposure to ground level ozone increases emergency department (ED) visit for cellulites. We measure this increase in the corresponding odds ratios.

## 2. Experimental Section

### 2.1. Study Population

The study sample included all patients served by 5 hospitals in Edmonton, Canada, during the period of 1 April 1992 to 31 March 2002 (2,946,714 ED visits were recorded). The hospitals provide complete health services to approximately one million residents in the greater Edmonton area and also to visitors to the region who experience a health emergency (~9% of all patients).

### 2.2. Environmental Data

Hourly air pollution levels for ozone were obtained from automated fixed-site monitoring stations in the National Air Pollution Surveillance Network maintained by Environment Canada. The daily mean ozone level was calculated as the average of 24 hourly measures in the same day at each station. The daily per-station data were averaged across the 3 monitoring stations operating in Edmonton urban area. Ozone concentrations were originally expressed in parts per billion (ppb) and, consequently, mean concentrations, standard deviation, median, and interquartile range are expressed in this same unit.

Environment Canada also supplied hourly data for relative humidity and temperature for the city of Edmonton. We calculated the daily levels of weather parameters, temperature and relative humidity, by averaging hourly data over 24-hour periods.

### 2.3. Health Outcomes

The investigated health outcome was ED visit for cellulitis. The term “cellulitis” as used in this study signifies inflammation of deep skin (*dermis* and subcutaneous tissues). *The International Classification of Diseases 9th Revision* (ICD-9) allocates codes 682.X, 681.00, 681.10, 686.9, or 958.3 to distinct cellulitis cases. In the records we obtained from Edmonton hospitals (release diagnoses), such skin disorders were described explicitly as “cellulitis” or implicitly as e.g., inflammation of subcutaneous tissue or post-traumatic skin infection, and assigned the ICD-9 codes.

Counts of the most frequent ED visits for a cellulitis case and the corresponding ICD-9 code are shown in Table 1. Less frequent visits for cellulitis cases described as e.g., “blister with infection” or “insect bite with infection”, were also included in the analysis. The count of all visits for cellulitis mounted to N = 69,547 (2.4% of all ED visits).

We also use term “restricted cellulitis” to distinguish cellulitis infection localized on face, neck, and upper limbs, which are often less covered than are the trunk and lower limbs (N = 31,851).

### 2.4. Statistical Analysis

Case-crossover (CC) designs are suitable for the estimation of odds of acute health effects triggered by acute and transient exposures [18]. The case crossover technique is an adaptation of case-control approach where the case serves as his/her own control. For each case of cellulitis, the case’s exposure to ozone at time of admission, or a lagged exposure, was compared to the exposure at referent time intervals (referent days). The referent days were selected in a way to match year, month, and day of week of the case day.

Conditional logistic regression model was used to estimate the odds ratio (OR) with its 95% confidence intervals (CI) associated with an increase in the interquartile range (14.0 ppb) of ozone after adjusting for temperature and relative humidity. In order to compensate for possibly non-linear weather effects, the models we constructed incorporated the temperature and relative humidity parameters in form of natural splines with 3 degrees of freedom.

The analysis was performed independently for specific age groups, for both sexes for all, warm and cold season (expressed by the corresponding months), and necessarily when applying 0- to 5-day lags to the exposure and weather parameters. Analysis was also stratified by location of cellulitis: air exposed (*i.e.*, face, neck, and upper limbs) *versus* less air exposed areas (*i.e.*, legs and trunk).

### 2.5. Ethics

Health Research Ethics Board of the University of Alberta approved this study protocol. The study was conceived and designed after 31 March 2002, *i.e.*, it is a retrospective study examining data produced between April 1992 and March 2002. In addition, Health Canada operationally approved the study, which makes use of de-identified patient data.

## 3. Results and Discussion

The meteorological factors, temperature and relative humidity, had the following characteristics; temperature (in Celsius degree): 3.9, 11.9, 5.4, and relative humidity (in percentage): 66.0, 13.6, 66.1, values for mean, standard deviation and median, respectively, The mean concentration of ground level ozone was found to be 18.6 ppb, with a standard deviation of 9.3, a median of 17.8, and an IQR of 14.0. These values apply to the average of measurements obtained from 3 monitoring sites in Edmonton: station northwest (N), central (C), and east (E). The correlation matrix for the measurements was found to be E-N: 0.85; E-C: 0.89; N-C: 0.88 (for the 3,552 days when all three monitors were operational). The average ozone level in warm months (April–September) was higher than in the cold months (October–March): 23.4 *vs.* 13.9.

Sunday and Monday are days of week when more visits happened, respectively (in %) 15.7 and 15.0. Least visits occurred on Thursday: 13.2. The weekly pattern recurred for the restricted cellulitis, with practically the same percentages.

August and July accounted for 10.3% and 10.1% of all ED visits for cellulitis, respectively, while the minimum was attained in January and February: 6.7% for each month. Also visits for restricted cellulitis peaked up in August (9.6%) and July (9.2%) and decreased in January (7.0%) and February (7.3%).

The most frequent ED patients with cellulitis were men and women 35–39 years old (11.4% of all visits for cellulitis), with men representing 66.8% of the age stratum. The same is true for the restricted cellulites (an uncovered skin area), with men making up a 67.9% of the stratum.

Table 2 shows the ORs and 95% CIs estimated for exposure data lagged by 0 to 4 days for ED patients (all, male, and female) diagnosed with cellulitis. The results suggest a correlation between the visits and a recent exposure (within the last 3 days for male patients and today’s or yesterday’s exposure for female patients). The effect is stronger in male subjects, due mainly to the positive and significant associations between the visit and 2- and 3-day old exposures in the cold season and 4-day old exposure in the warm season. No such effect was observed among female patients. Also positive and significant association can be observed between exposure and a next-day visit (for both male and female patients) and between exposure and same-day visit (for female patients only).

Table 3 shows estimates analogous to those in Table 2 for a reduced scope of health outcome: cases of cellulitis localized to mostly uncovered areas of skin. In this situation, the exposure effects were found to be generally stronger, and also more polarized along the ‘male patients in cold season— female patients in warm season’ axis.

Finally, it is to be noted that we have created a complementary table analogous to Table 3 but showing the results for cases of cellulitis localized in mostly covered skin areas (54.2% of all cellulitis cases). We further concluded from the almost complete absence of positive significant results that this health outcome did not show considerable dependency on the ozone exposure.

Table 4 illustrates, for selected groups of patients, ozone exposure effects as a function of patient age, expressed as ORs and CIs for cellulitis (in a broad sense) *vs.* ORs and CIs for restricted cellulitis. The effects are found to be generally stronger for the latter health outcome. The table also demonstrates a change in the effects when moving from a younger to an older group of patients. In particular, results for the cellulitis restricted to uncovered skin and to patients aged 66 or older are insignificant. For this age stratum, results were also insignificant when we analyzed the complementary case, *i.e.*, the cellulitis occurring in covered skin.

Table 4 does not list the group of female patients of age 2 or younger, where results for the general cellulitis or a specific cellulitis were not significant. For the same reason results for boys 3–11 and girls 12–14 are not presented. This lack of significance may be due to reduced dimensions of the strata. Also not all both-sexes strata are listed, seeing that the results for an age stratum just did reflect the results obtained for the one-sex refinement.

The age groups listed in the table were determined by sorting out sub-samples by moving age windows of various widths and determining the age-and-sex strata for which the estimates reached local maxima.

To our knowledge there is only one major epidemiological study linking human skin problems with ozone [19]. In this study, we detected positive and significant effects of ozone exposure on presentation to the emergency department for cellulitis. When the analysis was restricted to cellulitis occurring in regions with greater air exposure (*i.e.*, arms, face, and neck) the effects of ozone were strengthened. In contrast, cellulitis localized to the trunk and legs were not affected by acute ambient exposure to ozone.

The vast majority of ambient ground level ozone arises from combustion of fossil fuels (motor vehicles mainly) and as a polluting by-product of several heavy industries (rotating machinery, petrochemicals) in Edmonton. The city has one of the highest monthly averages of ozone levels, comparable to the averages in Toronto, Ontario, with a peak in May (~53 ppb) and a low in November (~9 ppb); however, the short-term variability of daily averages is comparatively small. Also, the daily averages of ozone mixing ratios in the suburban localities reflect those in the city [20], as a result of solar radiation flux at Edmonton’s latitude (>51°N) acting upon a stagnant air mass with urban inputs of ozone precursors [21].

When all cases were analyzed, the effect of ozone on cellulitis was not different between males and females, but the risk of ozone was greatest in warm months as compared to cold months. Ozone arises from emissions that react with sunlight [22], consequently ozone concentrations are highest in warm months. Furthermore, observations of greater effects of ozone exposure in warm seasons may be a consequence of a better correlation between the actual and the measured exposure as in warm weather individuals are outdoors more often. In contrast, when the analysis was restricted to cellulitis occurring sun exposed areas, differential effects were observed such that men were more likely to develop cellulitis in cold seasons and women during warm seasons. Cold weather, and associated with it dryness of skin, may be a factor increasing sensitivity to ozone in men. However, future studies will be necessary to confirm these findings and explain the mechanism of association.

Differential effects of ozone on cellulitis were observed across age groups. Infants and children were at increased risk for presenting to the ED with cellulitis. Children are more likely to be outside, particularly in warm seasons, as compared to adults, and thus may have greater exposure to ozone. An increased risk associated with ozone was observed in adult individuals of age 65 or younger, as aged or photo-aged skin is a risk factor for inflammation [23,24] that may be exacerbated by ozone exposure. The risk may diminish for the older-age group, as sensitivity to ozone in older individuals was observed to be low [5].

The mechanisms by which ozone may influence the development of cellulitis are speculative. Ozone may predispose to cellulitis infections through oxidative stress [25,26]. Ozone acting on exposed skin may breach the skins’ barrier function and allow bacteria to penetrate the deeper layers of skin, and/or worsening the symptoms of a pre-existing infection. Animal studies have demonstrated that ozone exposure may cause structural skin damage, which may increase the permeability of the skin to bacteria [27]. The reliability of this study is enhanced by adequate controlling for the non-linear meteorological confounders, temporal trends (long-term time effects, seasonal cycle, month-of-year impact), and the weekly cycle. Furthermore, the CC design has the inherent ability to control for time independent risk factors (e.g., patient’s diabetes). Also, a large sample size and a long study period ensure adequate statistical power required for evaluating the odds of ED visit for cellulitis in a group such as young children of one sex or for particular cases of cellulitis in a time stratum.

The results should be interpreted in the context of the limitations of the study. Cellulitis cases were identified by using ICD-9, which may result in misclassification of the diagnosis; however, random misclassification errors would bias our results towards the null. Next, regional estimates of ozone exposure may not have correlated with patient-level exposures prior to visits to the ED, which would also bias the risk estimates towards null. A classification of severity of cellulitis infection was unavailable, and milder cases, adequately controlled with oral antibiotics may not have been presented to emergency departments. Furthermore, some patients may have repeat visits to the emergency department; repeat admissions for follow-up would not be correlated to ozone concentrations in the air. While time-stable confounders (e.g., genetics) are controlled for by the case crossover study design, confounders that fluctuate in time may not have been controlled. Weather parameters (*i.e.*, temperature and humidity) were adjusted in the analysis; however, ultraviolet radiation was not. Ultraviolet radiation may synergistically aggravate skin inflammation, but elevated ultraviolet levels may increase the amount of ground level ozone and photochemical smog [28]. Also, the ultraviolet-induced oxidative stress shows close relationship to ozone-induced oxidation at the biochemical level [29,30]. Thus, future studies will be needed to replicate our findings and confirm that the increased risk of cellulitis was specifically due to ozone exposure [31].

## 4. Conclusions

This study suggests a novel modifiable risk factor for development of cellulitis among at risk populations. Increase in concentration of ambient ground-level ozone was associated with increased hazard of emergency department visit for cellulites in distinct age groups and in characteristic environmental conditions enhancing ozone potency Future epidemiological and basic science studies are necessary to replicate these findings and further evaluate the relationship between ambient ozone levels and cellulitis. If these findings are confirmed, some cases of cellulitis may be prevented by improving air quality or by encouraging susceptible populations to cover their skin.

## Figures and Tables

**Table 1:** Most frequent cases among all ED visits for cellulitis (N = 69,547).

	Codes	All		Male		Female	

Diagnosis Description	ICD-9	Count	% N	Count	% N	Count	% N
cellu/abscess leg exc foot	682.6	19,692	28.3	12,109	17.4	7,583	10.9
cellu/abscess upp/forearm *	682.3	11,076	15.9	7,710	11.1	3,366	4.8
cellu/abscess foot exc toe	682.7	7,012	10.1	4,351	6.3	2,661	3.8
cellu/absc hand exc finger *	682.4	6,743	9.7	4,611	6.6	2,132	3.1
cellu and abscess of face *	682.0	5,355	7.7	2,744	4.0	2,611	3.7
cellu/abscess finger nospec *	681.00	4,746	6.8	3,29	4.4	1,717	2.5
nspec local infect skin *	686.9	3,739	5.4	2,302	3.3	1,437	2.1
cellu and abscess of trunc	682.2	2,724	3.9	1,283	1.8	1,441	2.1
cellu and abscess toe nospec	681.10	2,190	3.2	1,370	2.0	820	1.2
posttrauma wound infect neck *	958.3	1,298	1.9	793	1.1	505	0.7

**Total**		**64,575**	**92.9**	**40,302**	**58.0**	**24,273**	**34.9**

* Diagnosis considered as cellulitis localized in uncovered area of skin.

**Table 2:** Odds ratio and its 95% CI, by sex, season, and exposure lag, for ED visits for cellulitis (N = 69,547).

		All Patients		Male		Female	

Season	Lag	OR	95% CI	OR	95% CI	OR	95% CI
All	0	1.05	1.02, 1.07 a	1.04	1.01, 1.07 a	1.05	1.01, 1.09 a
	1	1.05	1.03, 1.08 a	1.04	1.01, 1.07 a	1.08	1.04, 1.12 a
	2	1.05	1.02, 1.07 a	1.05	1.02, 1.08 a	1.04	1.01, 1.08 a
	3	1.03	1.01, 1.05 a	1.05	1.02, 1.08 a	1.00	0.96, 1.03
	4	1.02	0.99, 1.04	1.03	1.01, 1.06 a	0.99	0.96, 1.03
Warm	0	1.07	1.04, 1.11 a	1.04	0.99, 1.09	1.12	1.06, 1.18 a
	1	1.08	1.04, 1.12 a	1.05	1.01, 1.10 a	1.12	1.06, 1.19 a
	2	1.04	1.01, 1.08 a	1.04	0.99, 1.09	1.05	0.99, 1.11
	3	1.03	1.01, 1.07 a	1.04	0.99, 1.08	1.03	0.98, 1.09
	4	1.04	1.01, 1.08 a	1.06	1.01, 1.11 a	1.02	0.97, 1.08
Cold	0	1.02	0.99, 1.06	1.05	1.01, 1.09 a	0.99	0.94, 1.04
	1	1.03	0.99, 1.06	1.03	0.99, 1.07	1.03	0.98, 1.08
	2	1.04	1.01, 1.08 a	1.05	1.01, 1.09 a	1.03	0.98, 1.08
	3	1.02	0.99, 1.05	1.06	1.02, 1.10 a	0.96	0.92, 1.01
	4	0.99	0.96, 1.02	1.01	0.97, 1.05	0.96	0.92, 1.01

a p-value < 0.05.

**Table 3:** Odds ratio and its 95% CI, by sex, season, and exposure lag, for ED visits for cellulitis localized in an uncovered area of skin (N = 31,851).

		All Patients		Male		Female	

Seasons	Lag	OR	95% CI	OR	95% CI	OR	95% CI
All	0	1.10	1.06, 1.13 a	1.09	1.05, 1.14 a	1.11	1.05, 1.17 a
	1	1.09	1.06, 1.13 a	1.06	1.02, 1.11 a	1.15	1.09, 1.21 a
	2	1.07	1.04, 1.11 a	1.05	1.01, 1.09 a	1.12	1.06, 1.18 a
	3	1.03	0.99, 1.06	1.06	1.01, 1.10 a	0.99	0.93, 1.04
	4	1.00	0.96, 1.03	1.02	0.98, 1.07	0.95	0.90, 1.01
Warm	0	1.10	1.05, 1.16 a	1.05	0.99, 1.12	1.18	1.09, 1.28 a
	1	1.09	1.04, 1.15 a	1.03	0.96, 1.10	1.21	1.11, 1.32 a
	2	1.04	0.98, 1.09	0.98	0.91, 1.04	1.15	1.06, 1.26 a
	3	1.01	0.96, 1.07	1.00	0.93, 1.06	1.04	0.96, 1.14
	4	1.03	0.98, 1.08	1.03	0.96, 1.10	1.03	0.95, 1.12
Cold	0	1.09	1.04, 1.14 a	1.13	1.06, 1.19 a	1.02	0.95, 1.10
	1	1.09	1.04, 1.14 a	1.09	1.03, 1.15 a	1.08	1.01, 1.16 a
	2	1.09	1.04, 1.14 a	1.10	1.04, 1.16 a	1.07	1.01, 1.16 a
	3	1.04	0.99, 1.09	1.11	1.05, 1.17 a	0.93	0.86, 1.00
	4	0.97	0.93, 1.01	1.02	0.97, 1.08	0.88	0.82, 0.90^b^

a p-value < 0.05.

b p-value < 0.01

**Table 4:** Odds ratios and their 95% CIs for ED visits for cellulitis *vs.* cellulitis localized in uncovered skin area in selected groups of patients, by age and sex.

Patients Sex/Age		Exposure Lag	Cellulitis—Any Site			Cellulitis—Uncovered Site		
			n	OR	95% CI	n	OR	95% CI
M	0–2	0	597	1.35	1.05, 1.73 a	318	1.59	1.13, 2.24 a
		3		1.29	1.01, 1.66 a		1.42	0.99, 2.03
		4		1.40	1.10, 1.79 a		1.61	1.14, 2.26 a
		5		1.31	1.03, 1.66 a		1.42	1.02, 1.99 a

All	3–11	1	2,574	1.07	0.97, 1.19	1,312	1.22	1.03, 1.44 a
F	3–11	1	1,199	1.18	0.99, 1.41	614	1.32	1.03, 1.68 a

All	12–14	3	1,016	1.23	1.02, 1.49 a	487	1.36	1.03, 1.79 a
		4		1.28	1.06, 1.56 a		1.39	1.05, 1.85 a
		5		1.27	1.05, 1.53 a		1.49	1.12, 1.98 a
M	12–14	3	626	1.15	0.90, 1.47	314	1.53	1.09, 2.15 a
		4		1.23	0.97, 1.57		1.58	1.12, 2.22 a
		5		1.25	0.98, 1.59		1.73	1.21, 2.48 a

M	15–34	5	12,336	1.01	0.96, 1.07	7,100	1.07	1.01, 1.15 a
F	15–34	1	7,046	1.08	1.01, 1.16 a	3,285	1.15	1.03, 1.27 a
		2		1.02	0.95, 1.09		1.12	1.01, 1.25 a

M *	35–65	0	11,075	1.07	1.02, 1.13 a	5,150	1.19	1.10, 1.28 a
		1		1.06	1.01, 1.12 a		1.14	1.06, 1.23 a
		2		1.07	1.02, 1.13 a		1.08	1.01, 1.17 a
		3		1.09	1.03, 1.15 a		1.11	1.02, 1.19 a
F **	35–65	0	6,730	1.15	1.06, 1.25 a	2,920	1.29	1.14, 1.46 a
		1		1.17	1.08, 1.26 a		1.26	1.11, 1.43 a
		2		1.11	1.03, 1.21 a		1.25	1.11, 1.42 a
		3		1.05	0.97, 1.14		1.12	0.99, 1.27

M	66+older	0	5,343	1.08	1.01, 1.18 a	1,653	1.01	0.87, 1.17
		1		1.10	1.01, 1.20 a		1.10	0.95, 1.28
		2		1.04	0.96, 1.13		1.15	0.99, 1.33
F	66+ older	0	4,802	1.13	1.03, 1.23 a	1,410	1.12	0.96, 1.31
		1		1.09	1.01, 1.19 a		1.11	0.95, 1.30
		2		1.10	1.01, 1.20 a		1.12	0.96, 1.32
		3		1.17	1.08, 1.28 a		1.13	0.96, 1.33
		4		1.14	1.05, 1.24 a		1.09	0.93, 1.28

a p-value < 0.05.

* Cases in the cold months only (October–March).

** Cases in the warm months only (Apri–September). All: both sexes, M: males, F: females.
